# Combination of Active Components of Xiexin Decoction Ameliorates Renal Fibrosis Through the Inhibition of NF-κB and TGF-β1/Smad Pathways in db/db Diabetic Mice

**DOI:** 10.1371/journal.pone.0122661

**Published:** 2015-03-24

**Authors:** Jia-Sheng Wu, Rong Shi, Xiong Lu, Yue-Ming Ma, Neng-Neng Cheng

**Affiliations:** 1 Department of Pharmacology, Shanghai University of Traditional Chinese Medicine, Shanghai, China; 2 Experiment Center for Science and Technology, Shanghai University of Traditional Chinese Medicine, Shanghai, China; 3 Department of Pharmacology, School of Pharmacy, Fudan University, Shanghai, China; University of Louisville, UNITED STATES

## Abstract

Xiexin decoction, a herbal therapeutic agent commonly used in traditional Chinese medicine, is recognized for its beneficial effects on diabetic nephropathy exerted through the combined action of multiple components, including Rhizoma Coptidis alkaloids (A), Radix et Rhizoma Rhei polysaccharides (P), and Radix Scutellaria flavones (F). Our previous studies have shown that a combination of A, P, and F (APF) exhibits renoprotective effects against diabetic nephropathy. This study was aimed at determining the effects of APF on renal fibrosis in diabetic nephropathy and elucidating the underlying molecular mechanisms. To evaluate the effects of APF, in vivo, db/db diabetic mice were orally administered a low or high dose of APF (300 or 600 mg/kg, respectively) once a day for 8 weeks. We evaluated the blood and urine indices of metabolic and renal function, renal tissue histopathology, renal inflammation, and fibrosis. APF treatment significantly ameliorated glucose and lipid metabolism dysfunction, decreased urinary albumin excretion, normalized creatinine clearance, and reduced the morphological changes in renal tissue. Additionally, APF administration in db/db diabetic mice reduced the elevated levels of renal inflammation mediators such as intercellular adhesion molecule-1, monocyte chemotactic protein-1, tumor necrosis factor-α, interleukin-1β, and active nuclear factor κB (NF-κB). APF treatment also reduced type I and IV collagen, transforming growth factor-β1 (TGF-β1), and TGF-β1 type II receptor expression levels, and decreased the phosphorylation of Smad2/3 in the kidneys of db/db diabetic mice. These results suggest that APF reduces renal fibrosis in diabetic nephropathy through the NF-κB and TGF-β1/Smad signaling pathways. In vitro, APF treatment reduced cell proliferation and protein expression of α-smooth muscle actin, collagen I, TGF-β1 and NF-κB in mesangial cells cultured with high glucose concentrations. Our findings indicate that treatment with multi-component herbal therapeutic formulations may be a useful approach for the treatment of diabetic nephropathy.

## Introduction

Diabetes is a chronic metabolic syndrome and its vascular complications are responsible for morbidity and mortality in diabetic patients [1]. Diabetic nephropathy (DN) is a common complication of diabetes that leads to end-stage kidney failure [2]. Despite the widespread use of numerous therapeutic approaches focused on managing hyperglycemia and high blood pressure, high proportion of diabetic patients still suffer from progressive and severe renal injury [3–5]. Therefore, there is a critical need to develop novel renoprotective interventions for the treatment of DN.

The major pathological features of DN include mesangial expansion, extracellular matrix (ECM) alterations, tubulointerstitial fibrosis, and glomerular sclerosis [6]. Transforming growth factor-β1 (TGF-β1) has been identified as a key regulator of fibrosis in DN [7, 8]. Results from diabetic animal models and human patients have demonstrated that persistent hyperglycemia increases renal TGF-β1 expression [8, 9]. TGF-β1 exerts its effects by binding to the membrane-bound TGF-β1 type II receptor (TβRII), which activates TβRI kinase, resulting in the phosphorylation and activation of Smad2/3. Activated Smad2/3 proteins form oligomeric complexes with Smad4 proteins and translocate into the nucleus, where they induce the expression of target genes, including ECM proteins, contributing to the development of tubulointerstitial and glomerular fibrosis [10, 11]. Therefore, inhibiting TGF-β1/Smad pathway may have therapeutic potential against diabetic renal fibrosis. Several reports have suggested that the use of anti-TGF-β monoclonal antibody [12] and TGF-β receptor inhibitor [13] to block the TGF-β pathway could attenuate renal fibrosis in diabetic animal models, but may increase renal inflammation [13]. It has been shown that inflammation also plays an important role in the development of DN, and NF-κB is a critical signaling pathway to mediate the progress [14]. Therefore, alternative approaches that could inhibit NF-κB and TGF-β and protect against renal injury are needed. In China, traditional Chinese medicine (TCM) has a long history of use in the treatment of diabetes [15, 16], showing a number of unique advantages in the prevention of diabetic complications [17–19] over the conventional medical approach. There is great potential for identification of novel anti-DN drugs through the evaluation of the inhibition of NF-κB and TGF-β1/Smad signaling pathways by TCM agents.

Xiexin decoction (XXD) is a classic TCM prescription containing Radix et Rhizoma Rhei, Rhizoma Coptidis, and Radix Scutellaria. It has been used in the treatment of diabetes for 1300 years [20]. Our previous studies showed that XXD has beneficial effects on early-stage DN in rats [21, 22], but the potency of the compounded crude extract is somewhat limited. To improve the effectiveness and quality of the medicine, pharmacodynamic evaluations of the active constituents have been conducted, identifying Rhizoma Coptidis alkaloids (A), Radix et Rhizoma Rhei polysaccharides (P), and Radix Scutellaria flavones (F) among the main active constituents of XXD that are effective against DN [22, 23]. In addition, the combination of A, P, and F (APF) has a significant protective effect on renal injury in rats with high-fat diet and streptozotocin-induced DN, suggesting that APF may be more appropriate than XXD for widespread applications in patients. APF significantly inhibited renal inflammation and reduced TGF-β1 expression in DN rats. However, the effects of APF on renal injury in db/db mice, a murine model biochemically closest to the human type 2 diabetes, and mesangial cells incubated at high glucose are still unknown. Additionally, the effect of APF on renal fibrosis in DN and the associated mechanisms are also unclear. Therefore, the aim of the present study was to examine whether APF treatment has protective effects on diabetic renal fibrosis in vivo and in vitro and to explore the underlying molecular mechanisms.

## Materials and Methods

### Materials

Radix et Rhizoma Rhei (*Rheum palmatum* L.), Rhizoma Coptidis (*Coptis Chinensis* Franch), and Radix Scutellaria (*Scutellaria baicalensis* Georgi) were purchased from Shanghai Kang Qiao Herbal Pieces Co., Ltd (Shanghai, China) and carefully authenticated by Prof. Zhi-Li Zhao of the Department of Botany, Shanghai University of TCM. ELISA kit for measurements of serum insulin was obtained from R&D Systems (Minneapolis, MN, USA). Antibodies specific for inhibitor of nuclear factor-κB subunit α (IκBα), nuclear factor-κB (NF-κB), phospho-IκBα (ser32), phospho-nuclear factor-κB p65 (ser276), phospho-Smad2 (ser465), phospho-Smad3 (ser423), TGF-β1, and β-actin were purchased from Cell Signaling Technology, Inc. (Beverly, MA, USA). Antibodies specific for Smad7, monocyte chemotactic protein-1 (MCP-1), and intercellular adhesion molecule-1 (ICAM-1) were purchased from Santa Cruz Bio-technology (Santa-Cruz, CA, USA). Trizol, SuperScript cDNA Synthesis Kit, and Fast SYBR Green mix kit were purchased from Takara (Tokyo, Japan). 3-[4, 5-Dimethylthiazol-2-yl]-2, 5-diphenyltetrazolium bromide (MTT) was purchased from Sigma-Aldrich Chemical Co. (Saint Louis, MO, USA). Dulbecco’s modified Eagle’s minimum essential medium (DMEM) was obtained from Invitrogen (Carlsbad, CA, USA). Fetal bovine serum (FBS) was obtained from FuMeng Gene Co. Ltd. (Shanghai, China). The rat mesangial cell line (HBZY-1) was purchased from the Chinese Center for Type Culture Collection (Wuhan, China).

### Preparation and Quality Control of APF

APF was formulated in a 1:2:1 (A:P:F) ratio, with the preparation and quality control methods of these herbal components performed as previously described [24]. HPLC analysis of A and F was performed as previously reported [24] (see S1 Fig.). The mass percentages of berberine, coptisine, palmatine, and jatrorrhizine in A were determined to be 55.8, 8.5, 7.6, and 4.8%, respectively. The mass percentages of baicalin, wogonoside, and baicalein in F were 89.6, 0.1, and 3.0%, respectively. Polysaccharide content in P was determined to be 62.3% by using the phenol-sulfuric acid-UV method [24]. The monosaccharide components of P included rhamnose, pectinose, xylopyranose, mannitose, and glucose, which were detected by gas chromatography (see S1 Fig).

### Animal Experiments

All animal experiments were performed with the approval of the Institutional Animal Care and Use Committee of Shanghai University of Traditional Chinese Medicine (Approval Number: 2012012). Male mutant C57BLKs/J db/db mice (a mouse model of type 2 diabetes) and their normal littermates (db/m, wild-type) were purchased from Model Animal Research Center of Nanjing University (Nanjing, China).

Twelve-week-old mice were housed in an air-conditioned room at 22–24°C under a 12-h dark/light cycle, and were given food and water ad libitum. Fasting blood glucose (FBG) was measured by the glucose oxidase method. All db/db mice with fasting blood glucose levels above 11.1 mmol/L were divided into 4 groups as described below: Diabetic control (db/db mice); low dose of APF (APF 300 mg/kg, db/db mice receiving 300 mg/kg APF, containing 75, 150, and 75 mg/kg of A, P, and F, respectively); high dose of APF (APF 600 mg/kg, db/db mice receiving 600 mg/kg of APF, containing 150, 300, and 150 mg/kg of A, P, and F, respectively); and positive control (db/db mice receiving 200 mg/kg metformin). Eight age-matched db/m mice were used as normal controls (db/m). Diabetic and normal control groups were treated with the identical volume of vehicle (normal saline). All treatments were administered by intra-gastric gavage once a day, for 8 weeks.

### Metabolic Parameters, Urinary Albumin Excretion, and Renal Function Analyses

After 8 weeks of treatment, mice were placed in metabolic cages for 24-h urine collection prior to the sacrifice. Blood was drawn at the time of sacrifice. Urine albumin was measured by radioimmunoassay (Atom High Technology, Beijing, China). Serum insulin was measured using an ELISA kit and homeostasis model assessment of insulin resistance (HOMA-IR) was calculated. Serum levels of creatinine and triglyceride, as well as urine creatinine levels, were measured using a Hitachi 7080 Chemistry Analyzer (*Hitachi* Ltd., *Tokyo*, *Japan*). Creatinine clearance (C_Cr_) was calculated as previously reported [22]. The kidneys were removed and weighed, and samples dissected and frozen at -70°C until processed for western blot and RNA extraction. Remaining kidney portions were used for analysis by light and electron microscopy.

### Light Microscopy

Kidneys were fixed in 10% formaldehyde and embedded in paraffin. Sections (4-μm thick) were cut and stained with hematoxylin and eosin, Periodic Acid Schiff (PAS), or Masson’s modified trichrome histological stains. Stained sections were examined at 400× magnification by an observer blinded to the treatment group from which the tissue slices originated. The ratio of the mesangial matrix area to total glomerular area (M/G) in PAS-stained sections and ratio of area with collagen accumulation to total glomerular area in Masson trichrome-stained sections were determined using the Image-Pro Plus quantitative software as previously described (Pax-it; Paxcam, Villa Park, IL, USA) [25]. In each section, 20 glomeruli were randomly selected and positive signals within the selected glomerulus were highlighted and measured, with the positive area quantified as a percentage of the entire glomerulus [25].

### Electron Microscopy

The kidneys were fixed in 2.5% glutaraldehyde (0.2 M cacodylate buffer, pH 7.4) and embedded in epoxy resin. Ultrathin sections were double-stained with 1.25% uranium acetate and 0.4% lead citrate, and examined under the JEM100CX-α electron microscope (JEOL Ltd., Tokyo, Japan).

### Real-time PCR Analysis

Total RNA was extracted from kidneys using Trizol. cDNA was synthesized using SuperScript cDNA Synthesis Kit. Quantitative real-time PCR was performed using the Fast SYBR Green mix kit with the primers presented in Table 1 on the ABI-StepOnePlus Sequence Detection System (Applied Biosystems, CA, USA). The relative mRNA expression levels were calculated using the 2^-ΔΔCt^ method. The expression of GAPDH mRNA was used as the endogenous reference control.

**Table 1:** Nucleotide sequence of primers used in Real-time PCR.

	Forward nucleotide sequence 5’–3’	Reverse nucleotide sequence 5’–3’
IL-1β	CTTCAGGCAGGCAGTATCACTCAT	TCTAATGGGAACGTCACACACCAG
TNF-α	CATGAGCACAGAAA GCATGATCCG	AAGCAGGAATGAGAAGAGGCTGAG
TGF-β1	CAACAATTCCTGGCGTTACCTTGG	GAAAGCCCTGTATTCCGTCTCCTT
collagen I	TGCCGTGACCTCAAGATGTG	CACAAGCGTGCTGTAGGTGA
collagen IV	ATGCCCTTTCTCTTCTGCAA	GAAGGAATAGCCGATCCACA
TβRII	GGCTCTGGTACTCTGGGAAA	AATGGGGGCTCGTAATCCT
Smad7	TCAGGTGGCCGGATCTCA	GGTTGATCTTCCCGTAAGATTCA
GAPDH	CAGATCCACAACGGATATATTGGG	CATGACAACTTTGGCATTGTGG

### Western Blot Analysis

The kidneys were homogenized in a radioimmunoprecipitation assay buffer as previously described [22]. Proteins in the lysates were separated on a SDS-PAGE gel and electro-blotted onto nitrocellulose membranes. Membranes were blocked for 1 h in 5% non-fat milk and incubated overnight with primary antibodies at 4°C. Blots were then washed with Tris-buffered saline/0.1% (by volume) Tween-20 and incubated for 1 h with secondary antibodies. After washing, protein bands were detected using the FluorChem E image detection system (ProteinSimple, Santa Clara, CA, USA). β-Actin was used as a loading control. Densitometry analysis was performed and results were expressed as the integrated optical density relative to β-actin.

### Immunohistochemistry

After antigen retrieval and blocking, 4-μm-thick paraffin-embedded kidney sections were incubated overnight at 4°C with anti-collagen I (Santa Cruz Biotechnology, Glostrup, Denmark), anti-collagen IV (Santa Cruz Biotechnology, Glostrup, Denmark), or anti-TGF-β1 primary antibodies; the specific staining was subsequently detected using the labeled streptavidin biotin+ system horseradish peroxidase (Dako, Glostrup, Denmark). A negative control was included in which the primary antibody was preincubated with a control peptide. Sections were examined using an Olympus DP72 microscope (Olympus, Tokyo, Japan) and digitized with a high-resolution camera (magnification, ×400). Glomeruli or tubulo-interstitial areas (n = 20, each) were randomly selected from each section and immunostaining was performed to detect TGF-β1, collagen I, and collagen IV. Immunostaining signals within each selected glomerular or tubulo-interstitial area were highlighted and quantified using Image-Pro Plus quantitative software as described above (Pax-it; Paxcam, Villa Park, IL, USA) [25]. Areas with positive staining were quantified and expressed as a percentage of the entire glomerulus or selected tubulo-interstitial area.

### Cell Culture

Mesangial cells were cultured in normal DMEM supplemented with 10% FBS, 2 mM glutamine, 100 U/mL penicillin, and 100 μg /mL streptomycin at 37°C in an atmosphere containing 5% CO_2_.

### Influence of APF on Proliferation of Mesangial Cells in High Glucose

Mesangial cells were seeded in 96-well plates at a density of 5 × 10^3^ cells /well with DMEM containing 5.5 mM glucose and 10% FBS. After incubation for 24 h, cells were further incubated with normal glucose levels (5.5 mM glucose), or high glucose levels (30 mM glucose). Cells incubated with high glucose levels were concurrently treated with a range of concentrations of APF (4, 8, or 16 μg /mL APF with 1:2:1 (A: P: F) ratio) or 2 mM metformin. After 24 h of treatment, cell proliferation was determined in each treatment group by the MTT test, performed as previously described [26].

### Effect of APF on Fibrosis in Mesangial Cells incubated in High Glucose

Mesangial cells were seeded in 24-well plates at a density of 5 × 10^4^ cells /well. After 24-h incubation, cells were further incubated with normal or high glucose concentrations, as described above. Cells incubated with high glucose levels were concurrently treated with a range of concentrations of APF (4, 8, or 16 μg/mL APF with 1:2:1 (A: P: F) ratio) or 2 mM metformin. After 24-h or 48-h of incubation, the cells were lysed with RIPA lysis buffer containing PMSF protease inhibitors. Protein concentrations were determined using the BCA method (Beyotime Institute of Biotechnology, China). The protein expression of α-smooth muscle actin (α-SMA), collagen I, TGF-β1 and NF-κB [25, 27–28] was measured by Western blot assay as above described.

### Statistical Analysis

Data are expressed as mean ± S.D. Statistical differences between the groups were evaluated by one-way analysis of variance (ANOVA). The dose-dependent nature of the relationship was evaluated by linear correlation analysis. A p-value of <0.05 was considered significant, and p < 0.01 was considered highly significant.

## Results

### Effects of APF on Glucose and Lipid Metabolism

At the end of the experiment, control db/db mice exhibited higher gain in body and kidney weights compared to the wild type (db/m) mice, while treatment with high dose of APF significantly decreased the kidney weight (Table 2). Additionally, the levels of FBG, serum insulin, HOMA-IR, and serum triglycerides were also markedly increased in control untreated db/db mice compared to the corresponding values in db/m mice. Treatments with a high dose of APF or metformin significantly lowered the levels of FBG, serum insulin, HOMA-IR, and serum triglycerides (Table 2).

**Table 2:** Effect of a combination of Rhizoma Coptidis alkaloids, Radix et Rhizoma Rhei polysaccharides, and Radix Scutellaria flavones (APF) on glucose and lipid metabolism, urinary albumin excretion and kidney function and renal function in db/db mice.

Groups	db/m	db/db	APF 300mg/kg	APF 600mg/kg	Metformin
Body weight (g)	26.0 ± 2.0**	47.0 ± 6.0	39.0 ± 5.0	41.0 ± 4.0	44.0 ± 6.0
Right Kidney weight (g)	0.20 ± 0.03*	0.23 ± 0.03	0.21 ± 0.02	0.18 ± 0.03**	0.21 ± 0.03
Fasting blood glucose (mmol/L)	7.6 ± 0.3**	29.3 ± 3.8	27.2 ± 4.2	22.0 ± 3.4**	18.1 ± 2.8**
Serum insulin (ng/ml)	2.0 ± 0.2**	8.2 ± 1.5	6.1 ± 0.6*	5.2 ± 0.5**	5.0 ± 0.4**
Serum TG (mmol/L)	0.44 ± 0.09**	1.11 ± 0.33	0.72 ± 0.10*	0.54 ± 0.22**	0.61 ± 0.16**
Home-IR	0.7 ± 0.1**	10.8 ± 2.5	7.4 ± 1.6*	5.1 ± 1.0**	4.1 ± 0.7**
Creatinine clearance (ml min^-1^kg^-1^)	7.4 ± 2.8**	3.7 ± 1.3	6.7 ± 1.4**	6.6 ± 1.9**	6.3 ± 3.3*
BUN (mmol/L)	8.5 1.0*	11.1 3.7	7.2 1.8**	7.4 0.8**	8.2 2.6**
Urinary albumin excretion (mg/day)	0.27 ± 0.06**	2.00 ± 0.57	1.35 ± 0.18**	0.91 ± 0.11**	1.02 ± 0.13**

Data are expressed as mean ±S.D., n = 10

*p<0.05

**p<0.01 as compared with db/db group

### Effects of APF on Urinary Albumin Excretion and Kidney Function

As summarized in Table 2, the 24-h urinary albumin excretion (UAE) was notably elevated in control db/db mice, as compared to the db/m mice. APF treatments significantly decreased urinary albumin excretion in a dose-dependent manner. Similarly, C_Cr_ was markedly decreased in control db/db mice in comparison to the db/m mice, and treatments with both doses of APF normalized C_Cr_.

### Effect of APF on Renal Histopathology and Ultrastructural Pathology

**Fig 1:**
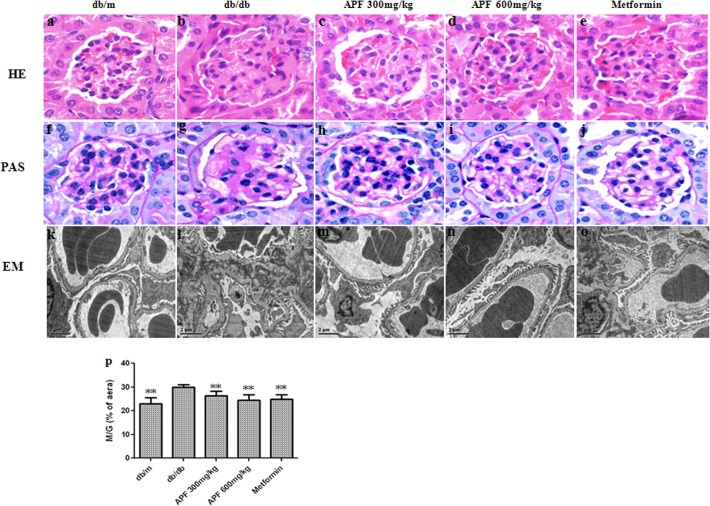
Effect of a combination of Rhizoma Coptidis alkaloids, Radix et Rhizoma Rhei polysaccharides, and Radix Scutellaria flavones (APF) on renal histopathology and ultrastructural pathology. (a-e) hematoxylin and eosin (HE) stain. (f-j) Periodic Acid Schiff (PAS) stain. Original magnification (a–j) × 400. (k-o) Electron microscopy (EM) analysis, Representative images of glomerular basement membrane thickening and mesangial matrix expansion, scale bars 2 μm, original magnification electron microscopy × 6000. (p) Ratio of the mesangial matrix area to total glomerular area (M/G) in PAS staining. Data are expressed as mean ± S.D., n = 10, **p < 0.01 as compared with db/db group.

Both hematoxylin and eosin and PAS staining demonstrated that compared to the db/m mice, the db/db mice had notable glomerular hypertrophy and mesangial matrix expansion (Fig. 1). Treatments with APF or metformin significantly ameliorated those changes. Additionally, PAS staining showed that M/G ratios were higher in db/db mice than in db/m mice, whereas treatments with APF and metformin significantly lowered the M/G ratio (Fig. 1). Electron microscopy of glomerular ultrastructure revealed mesangial expansion, mesangial matrix deposition, and glomerular basement membrane thickening in db/db mice (Fig. 1). However, these changes were significantly alleviated in APF and metformin treated db/db mice, compared with the control db/db mice (Fig. 1).

### Effect of APF on Renal Inflammation in db/db Mice

**Fig 2:**
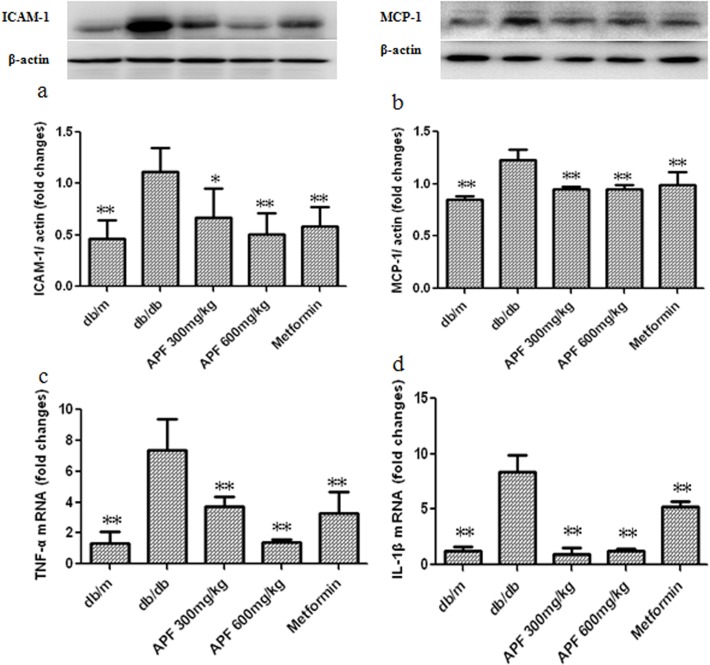
Effect of a combination of Rhizoma Coptidis alkaloids, Radix et Rhizoma Rhei polysaccharides, and Radix Scutellaria flavones (APF) on renal inflammation in db/db mice. (a-b) Western blot analysis of protein levels; (c-d) Real-time RCR analysis of mRNA levels; ICAM-1: intercellular adhesion molecule-1; MCP-1: monocyte chemotactic protein-1; TNF-α: tumor necrosis factor-α; IL-1β: interleukin-1β. Data are expressed as mean ±S.D., n = 3 for Western blot, and n = 5 for Real-time PCR, *p<0.05, **p<0.01 as compared with db/db group.

Since inflammation is a critical process in the development of fibrosis in DN, we examined whether APF treatment affects renal inflammation under diabetic conditions using real-time PCR and western blots. As shown in Fig. 2, renal expression of ICAM-1 and MCP-1 proteins, and levels of TNF-а and IL-1β mRNA were significantly increased in db/db mice. Treatments with both doses of APF normalized ICAM-1, and MCP-1 protein expression, and TNF-а and IL-1β mRNA levels.

### Effect of APF on Renal NF-κB Signaling Pathway

**Fig 3:**
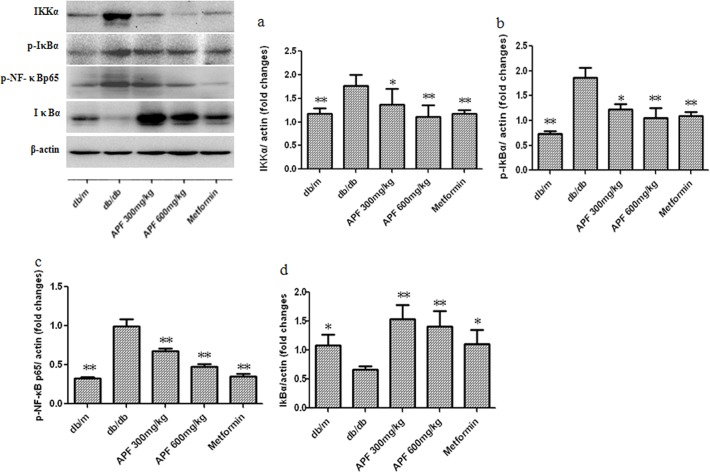
Effect of a combination of Rhizoma Coptidis alkaloids, Radix et Rhizoma Rhei polysaccharides, and Radix Scutellaria flavones (APF) on renal nuclear factor-κB (NF-κB) signaling pathway. IKKα: inhibitor of nuclear factor-κB kinase subunit α; IκBα, inhibitor of nuclear factor-κB subunit α; p-IκBα: phospho-IκBα; NF-κBp65: nuclear factor-κBp65; Data are expressed as mean ±S.D., n = 3, *p<0.05, **p<0.01 as compared with db/db group

Since NF-κB signaling pathway plays a key role in renal inflammation in DN, we investigated whether APF affects renal NF-κB signaling in db/db mice. As shown in Fig. 3, our data showed that compared to the db/m mice, the control db/db mice showed increased expression of renal IKKα, phospho-IκBα, and phospho-NF-κBp65 proteins with decreased IκBα expression. After 8 weeks of treatment, treatments with both doses of APF, as well as metformin, significantly ameliorated these alterations (Fig. 3).

### Effect of APF on Renal Fibrosis

**Fig 4:**
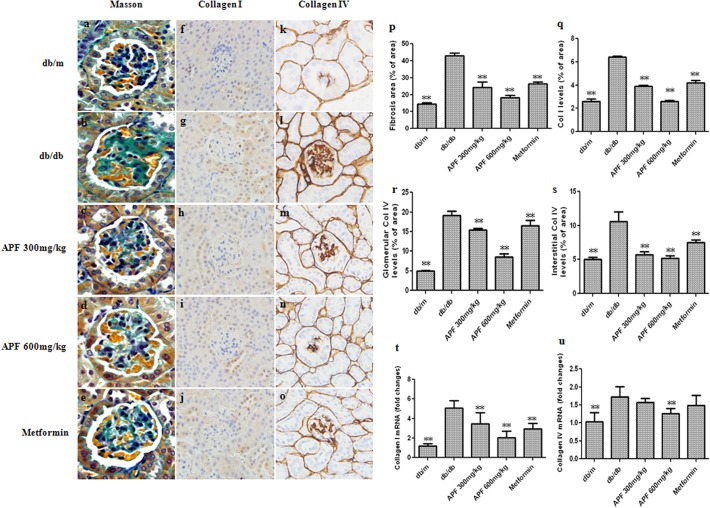
Effect of a combination of Rhizoma Coptidis alkaloids, Radix et Rhizoma Rhei polysaccharides, and Radix Scutellaria flavones (APF) on renal fibrosis. (a-e) Masson’s modified trichrome histological (Masson); (p) Ratio of area with collagen accumulation to total glomerular area; (f-j) Immunohistochemistry of collagen I; (k-o) Immunohistochemistry of collagen IV. Original magnification (a–o) × 400; (q) Quantitative analysis of immunohistochemical staining of collagen I (Col I); (r) glomerular of collagen IV (Col IV); (s) interstitial of collagen IV (Col IV); (t-u) Real-time RCR analysis of collagen I and collagen IV mRNA levels. Data are expressed as mean ±S.D., n = 5, **p<0.01 as compared with db/db group.

As presented in Fig. 4, Masson’s trichrome staining in renal tissues was performed to assess regional changes in collagen accumulation in diabetic kidneys. The results showed notable deposition of collagen in glomerular areas of db/db mice, while treatments with APF effectively prevented collagen deposition (Fig. 4). Additionally, protein and mRNA expression of collagen I and collagen IV were notably elevated in control db/db mice, as compared with the db/m mice (Fig. 4). However, treatment with APF significantly ameliorated these changes in a dose-dependent manner (Fig. 4).

### Effect of APF on Renal TGF-β1 and its Receptor Expression

**Fig 5:**
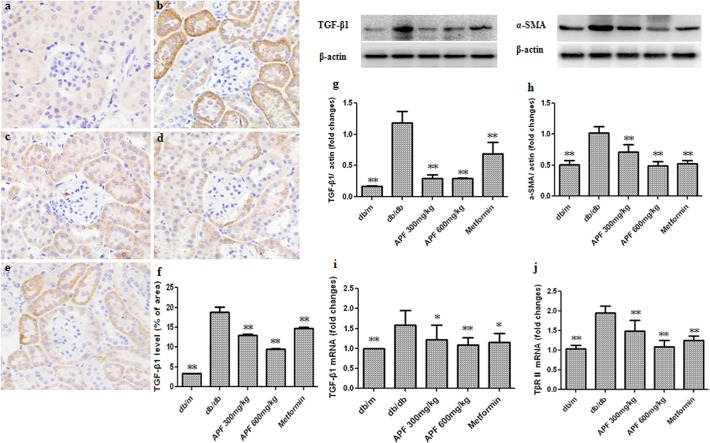
Effect of a combination of Rhizoma Coptidis alkaloids, Radix et Rhizoma Rhei polysaccharides, and Radix Scutellaria flavones (APF) on renal TGF-β1 and its receptor expression. (a-e) Immunohistochemistry of TGF-β1; (f) Quantitative analysis of immunohistochemical staining of TGF-β1; (g-h) Western blot analysis of TGF-β1 and α-SMA protein levels; (i-j) Real-time RCR analysis of TGF-β1 and TβRⅡmRNA levels. a: db/m, b: db/db, c: APF 300mg/kg, d: 600 mg/kg, e: metformin. Data are expressed as mean ±S.D., n = 3 for Western blot, and n = 5 for Immunohistochemistry and Real-time PCR, *p<0.05, **p<0.01 as compared with db/db group.

TGF-β1 has been identified as a key regulator of renal fibrosis in DN. To elucidate the mechanisms by which APF inhibits renal fibrosis, we examined the effects of APF on the expression of TGF-β1 and TβR II in diabetic kidneys using real-time PCR and western blot. As shown in Fig. 5, our data showed that compared to the db/m mice, the control db/db mice showed increased renal expression of TGF-β1 protein and mRNA, with up-regulation of TβR II protein expression. Significantly decreased renal expression of TGF-x1 and TβR II was observed after 8 weeks of treatment with both doses of APF or metformin (Fig. 5).

### Effect of APF on Renal TGF-β1/Smad Signaling Pathway

**Fig 6:**
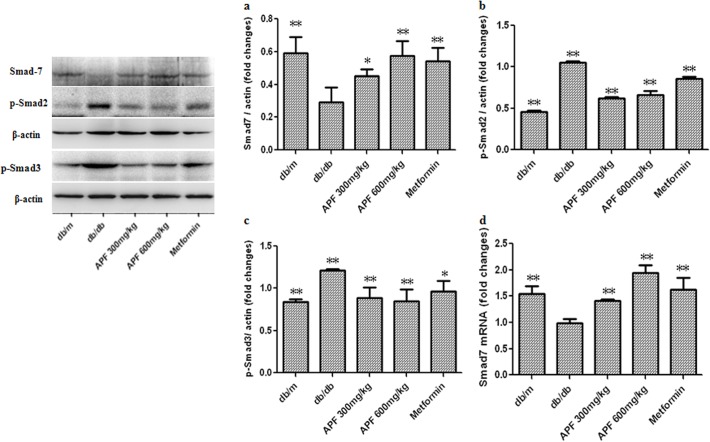
Effect of a combination of Rhizoma Coptidis alkaloids, Radix et Rhizoma Rhei polysaccharides, and Radix Scutellaria flavones (APF) on renal TGF-β1/Smad signaling pathway. (a-c) Western blot analysis of Smad7, p-Smad3 and p-Smad2 protein levels; d) Real-time RCR analysis of Smad7 mRNA level. Data are expressed as mean ±S.D., n = 3 for Western blot, and n = 5 for Real-time PCR, *p<0.05, **p<0.01 as compared with db/db group.

Activation of the Smad pathway and its subsequent nuclear translocation are critical steps in TGF-β1-mediated renal fibrosis in DN. As shown in Fig. 6, increased renal expression of TGF-β1 resulted in a significant increase in Smad2 and Smad3 phosphorylation in the control db/db mice. Renal protein and mRNA expression levels of Smad7 were notably lower in the control db/db mice than in the db/m mice. Administration of APF for 8 weeks markedly inhibited the phosphorylation of Smad2/3, and increased Smad7 protein and mRNA expression in the kidneys of db/db mice (Fig. 6).

### Effects of APF on Cell Proliferation and Fibrosis in Mesangial cells incubated at High Glucose

**Fig 7:**
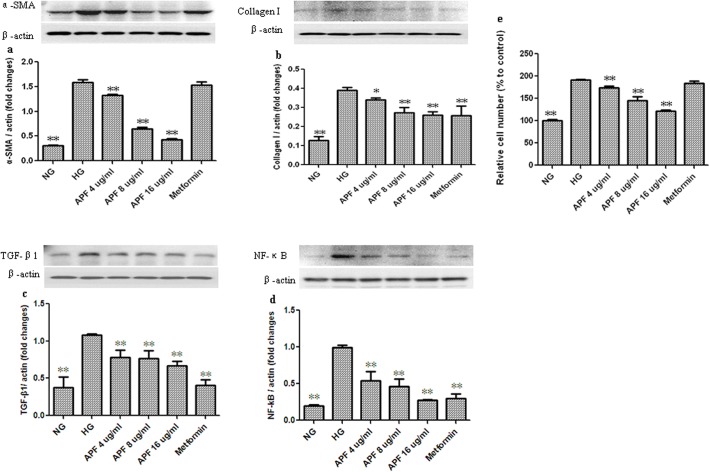
Effects of a combination of Rhizoma Coptidis alkaloids, Radix et Rhizoma Rhei polysaccharides, and Radix Scutellaria flavones (APF) on cell proliferation and fibrosis in mesangial cells incubated at high glucose. NG: normal glucose; HG: high glucose; APF 4, 8, and 16 μg /mL represent mesangial cells incubated in high glucose concentration treated with 4, 8, and 16 μg /mL APF, respectively. (a-d) Western blot analysis of α-SMA, CollagenⅠ, TGF-β1 and NF-κB protein levels; (e) Cell proliferation. Data are expressed as mean ±S.D., n = 5 for cell proliferation, and n = 3 for Western blot, *p<0.05, **p<0.01, compared to control cells incubated at high glucose concentration with no APF.

The proliferation of the mesangial cells in high-glucose conditions was significantly higher than that of cells incubated at normal glucose concentration (Fig. 7). However, compared with control cells incubated at high glucose concentration without any concurrent treatment, APF treatment (r = -0.970, p < 0.01) significantly inhibited cell proliferation in a dose-dependent manner (Fig. 7). Additionally, the expression of α-SMA, collagen I, TGF-β1 and NF-κB proteins in cells incubated at high glucose concentration was markedly increased compared with the cells incubated at normal glucose levels (Fig. 7), and treatment with APF significantly decreased those protein levels (Fig. 7).

## Discussion

Traditional herbal compounds have been widely used in the treatment of diabetes in China and are recognized as effective alternatives to conventional medicine [18, 29]. In our current study using a db/db mouse model of type 2 diabetes, we demonstrate that APF treatment has potential for use in the amelioration of glucose and lipid metabolism disorders, and can decrease urinary albumin excretion. Additionally, APF treatment normalized C_Cr_, and alleviated mesangial matrix expansion and glomerular basement thickening in db/db mice. These results suggest that APF has significant protective effects against diabetic renal injury. Further study revealed that APF reduced collagen accumulation both in the tubular interstitium and in the glomerular tissue of db/db mice. Consistent with the in vivo study, APF treatment decreased the expression of α-SMA and collagen I in mesangial cells incubated at high glucose levels. Taken together, these findings suggest that APF could protect against the progression of diabetic renal fibrosis.

TGF-β1 was confirmed to play a central role in a variety of fibrogenic processes, including ECM protein accumulation and tubulointerstitial and glomerular fibrosis [11]. In vitro and in vivo studies have revealed TGF-β1 to be an important mediator of renal fibrosis in DN [7, 30]. TGF-β1 may exert its effects through the Smad signaling pathways [30]. In diabetes, a number of factors could activate the TGF-β1/Smad signaling pathway, which may stimulate collagen matrix deposition and promote tubulointerstitial and glomerular fibrosis [30]. The results of our current study indicate that the expression of TGF-β1 protein was markedly upregulated in the kidneys of db/db mice and in the mesangial cells incubated at high glucose, and treatment with APF suppresses TGF-β1 expression. Additionally, APF treatment was shown to decrease TβRII expression and phosphorylation of Smad2/3 while increasing Smad7 expression in the kidneys of db/db diabetic mice. Moreover, APF treatment also markedly inhibited the protein and mRNA expression of collagen I and collagen IV in db/db diabetic mice kidney. These results suggest that APF negatively regulates the TGF-β1/Smad pathway to control renal fibrosis in DN.

Growing body of evidence suggests that inflammation plays an important role in diabetic kidney fibrosis [14, 31]. NF-κB signaling pathway is a critical component of renal inflammation in the progression of DN [32, 33]. In our current study, increased IκBa expression and decreased phosphorylation of NF-κB were observed in the kidneys of APF-treated db/db mice. In the mesangial cells incubated at high glucose, increased NF-κB expression was also decreased by APF treatment. Moreover, in our study we observed that alterations in a number of indices of renal inflammation, such as increased ICAM-1, MCP-1, TNF-а, and IL-1β expression in db/db diabetic mice, were alleviated by APF treatment. These results suggest that inhibition of the NF-κB signaling pathway and its effect on inflammation may be another key mechanism by which APF attenuates renal fibrosis.

The pathogenesis of DN is complicated. It was previously reported that inhibition of TGF-β activity alone may be associated with a number of adverse effects. For example, though anti-TGF-β monoclonal antibody [11] and TGF-β receptor inhibitor [12] were shown to inhibit the activity of TGF-β and may prevent glomerulosclerosis in some mouse models of DN, but also stimulate the renal inflammatory response [13]. Therefore, alternative approaches that can be used to inhibit TGF-β action and protect against renal inflammatory injury are needed. In our current study, APF treatment was found not only to inhibit TGF-β1/Smad-mediated fibrosis, but also to reduce NF-κB-dependent inflammation. These results suggest that APF, a mixture comprising multiple herbal compounds, may act on a number of targets simultaneously to generate a combined comprehensive effect, which may exhibit distinct advantages for treatment of DN compared to agents specific for a single molecular target.

As a multi-component herbal mixture, APF can be prepared using standardized methods and under strict quality control, thereby presenting a more appropriate therapeutic agent for broad application than the original TCM decoction. However, the evidence showing the ability of APF to ameliorate renal fibrosis, obtained from studies in animal and cell models, needs to be confirmed with further research in human diabetic patients.

In summary, our current study suggests that APF can inhibit renal fibrosis that is associated with DN by inhibiting the NF-κB and TGF-β1/Smad signaling pathways. Use of multiple herbal compounds may be a useful approach for treating DN.

## Supporting Information

S1 Fig Chromatograms of Rhizoma coptidis alkaloids (A), Radix Scutellaria flavones (F) and Radix et Rhizoma Rhei polysaccharides (P). A and F were detected by HPLC. P was detected by GC.
(TIF)

## References

[1] Sculy T. Diabetes in numbers. Nature. 2011; 485: S2–S3. 10.1038/485s2a 22616094

[2] Van Buren PN, Toto R. Current update in the management of diabetic nephropathy. Curr Diabetes Rev. 2013; 9: 62–77. 23167665

[3] Heerspink HJ, de Zeeuw D. The kidney in type 2 diabetes therapy. Rev Diabet Stud. 2011; 8: 392–402. 10.1900/RDS.2011.8.392 22262076 PMC3280673

[4] Yamout H, Lazich I, Bakris GL. Blood pressure, hypertension, RAAS blockade, and drug therapy in diabetic kidney. Adv Chronic Kidney Dis. 2014; 21: 281–286. 10.1053/j.ackd.2014.03.005 24780456

[5] Fried LF, Emanuele N, Zhang JH, Brophy M, Conner TA, Duckworth W, et al Combined angiotensin inhibition for the treatment of diabetic nephropathy. N Engl J Med. 2013; 369: 1892–1903. 10.1056/NEJMoa1303154 24206457

[6] Ziyadeh FN, Wolf G. Pathogenesis of the podocytopathy and proteinuria in diabetic glomerulopathy. Curr Diabetes Rev. 2008; 4: 39–45. 18220694 10.2174/157339908783502370

[7] Ziyadeh FN. Mediators of diabetic renal disease: the case for tgf-Beta as the major mediator. J Am Soc Nephrol. 2004; 15: S55–57. 14684674 10.1097/01.asn.0000093460.24823.5b

[8] Deshpande SD, Putta S, Wang M, Lai JY, Bitzer M, Nelson RG, et al Transforming growth factor-β-induced cross talk between p53 and a microRNA in the pathogenesis of diabetic nephropathy. Diabetes. 2013; 62: 3151–3162. 10.2337/db13-0305 23649518 PMC3749352

[9] Zorena K, Raczyńska D, Wiśniewski P, Malinowska E, Myśliwiec M, Raczyńska K, et al. Relationship between serum transforming growth factor beta 1 concentrations and the duration of type 1 diabetes mellitus in children and adolescents. Mediators Inflamm. 2013; 849457: 9. 10.1155/2013/849457 PMC3810184 24222720

[10] Huang C, Shen S, Ma Q, Chen J, Gill A, Pollock CA, et al Blockade of KCa3.1 ameliorates renal fibrosis through the TGF-β1/Smad pathway in diabetic mice. Diabetes. 2013; 62: 2923–2934 10.2337/db13-0135 23656889 PMC3717839

[11] Lan HY. Diverse roles of TGF-beta/Smads in renal fibrosis and inflammation. Int J Biol Sci. 2011; 7: 1056–1067. 21927575 10.7150/ijbs.7.1056 PMC3174390

[12] Ma LJ, Jha S, Ling H, Pozzi A, Ledbetter S, Fogo AB. Divergent effects of low versus high dose anti-TGF-beta antibody in puromycin aminonucleoside nephropathy in rats. Kidney Int. 2004; 65: 106–115. 14675041 10.1111/j.1523-1755.2004.00381.x

[13] Petersen M, Thorikay M, Deckers M, van Dinther M, Grygielko ET, Gellibert F et al Oral administration of GW788388, an inhibitor of TGF-beta type I and II receptor kinases, decreases renal fibrosis. Kidney Int. 2008; 73: 705–715 18075500 10.1038/sj.ki.5002717

[14] Navarro-González JF, Mora-Fernández C, Muros de Fuentes M, García-Pérez J.Inflammatory molecules and pathways in the pathogenesis of diabetic nephropathy. Nat Rev Nephrol. 2011; 7: 327–340. 10.1038/nrneph.2011.51 21537349

[15] Tong XL, Dong L, Chen L, Zhen Z. Treatment of diabetes using traditional Chinese medicine: past, present and future. Am J Chin Med. 2012; 40: 877–886. 10.1142/S0192415X12500656 22928822

[16] Xie W, Zhao Y, Zhang Y. Traditional chinese medicines in treatment of patients with type 2 diabetes mellitus. Evid Based Complement Alternat Med.2011; 2011: 726723 10.1155/2011/726723 21584252 PMC3092648

[17] Shi X, Lu XG, Zhan LB, Qi X, Liang LN, Hu SY, et al The effects of the Chinese medicine ZiBu PiYin recipe on the hippocampus in a rat model of diabetes-associated cognitive decline: a proteomic analysis. Diabetologia. 2011; 54: 1888–1899. 10.1007/s00125-011-2147-z 21509442

[18] Zhao HL, Sui Y, Qiao CF, Yip KY, Leung RK, Tsui SK, et al Sustained antidiabetic effects of a berberine-containing Chinese herbal medicine through regulation of hepatic gene expression. Diabetes. 2012; 61: 933–943. 10.2337/db11-1164 22396199 PMC3314348

[19] Wen X, Zeng Y, Liu L, Zhang H, Xu W, Li N, et al Zhenqing recipe alleviates diabetic nephropathy in experimental type 2 diabetic rats through suppression of SREBP-1c. J Ethnopharmacol. 2012; 142: 144–150. 10.1016/j.jep.2012.04.028 22564814

[20] Sun SM, (Tong Dynasty). Bei ji qian jin yao fang, Ancient Books Press of Traditional Chinese Medicine. 2009: 33–35.

[21] Wu JS, Lu X, Ma YM, Zhang N. Effect of Xiexin Decoction on early diabetic nephropathy in diabetic rats. Chin Tradit Herbal Drugs. 2010; 41: 73–77

[22] Wu JS, Shi R, Zhong J, Lu X, Ma BL, Wang TM, et al Renal protective role of Xiexin decoction with multiple active ingredients involves inhibition of inflammation through downregulation of the nuclear factor-κB pathway in diabetic rats. Evid Based Complement Alternat Med. 2013; 2013: 715671 10.1155/2013/715671 23935673 PMC3713598

[23] Liu Y, Cheng NN, Wu JS, Ma YM. Protective effects of ingredients of Xiexin Decoction on mesangial cells under high glucose condition. Chin J Clin Pharmacol. 2012; 21: 75–78

[24] Xie Y, Hu Y, Shen M, Ma Y, Zhong J, Zhang N, et al Dissolution and pharmacokinetic properties of alkaloids and flavonoids in a Xiexin multiple-unit drug delivery system. Drug Res (Stuttg). 2013; 63: 501–509. 10.1055/s-0033-1345206 23756913

[25] Ka SM, Yeh YC, Huang XR, Chao TK, Hung YJ, Yu CP, et al Kidney-targeting Smad7 gene transfer inhibits renal TGF-β/MAD homologue (SMAD) and nuclear factor κB (NF-κB) signalling pathways, and improves diabetic nephropathy in mice. Diabetologia. 2012; 55: 509–519. 10.1007/s00125-011-2364-5 22086159

[26] Gao J, Wang F, Wang W, Su Z, Guo C, Gao S. Emodin suppresses hyperglycemia-induced proliferation and fibronectin expression in mesangial cells via inhibiting cFLIP. PLoS One. 2014; Apr 1; 9 (4). 10.1371/journal.pone.0093588 PMC3972111 24691542

[27] Niu H, Nie L, Liu M, Chi Y, Zhang T, Li Y. Benazepril affects integrin- linked kinase and smooth muscle α-actin expression in diabetic rat glomerulus and cultured mesangial cells. BMC Nephrol. 2014; 15: 135 10.1186/1471-2369-15-135 25142208 PMC4151867

[28] Zhong X, Chung AC, Chen HY, Dong Y, Meng XM, Li R, et al miR-21 is a key therapeutic target for renal injury in a mouse model of type 2 diabetes. Diabetologia. 2013; 56: 663–674. 10.1007/s00125-012-2804-x 23292313

[29] Zhao TT, Zhang HJ, Lu XG, Huang XR, Zhang WK, Wang H, et al Chaihuang-Yishen granule inhibits diabetic kidney disease in rats through blocking TGF-β/Smad3 signaling. PLoS One. 2014; Mar 19; 9 (3) 10.1371/journal.pone.0090807 PMC3960111 24646636

[30] Lan HY. Transforming growth factor-beta/Smad signalling in diabetic nephropathy. Clin Exp Pharmacol Physiol. 2012; 39: 731–738. 10.1111/j.1440-1681.2011.05663.x 22211842

[31] Chow FY, Nikolic-Paterson DJ, Ozols E, Atkins RC, Tesch GH. Intercellular adhesion molecule-1 deficiency is protective against nephropathy in type 2 diabetic db/db mice. J Am Soc Nephrol. 2005; 16: 1711–1722. 15857924 10.1681/ASN.2004070612

[32] Yi B, Hu X, Zhang H, Huang J, Liu J, Hu J, et al Nuclear NF-kappaB p65 in peripheral blood mononuclear cells correlates with urinary MCP-1, RANTES and the severity of type 2 diabetic nephropathy. PLoS One. 2014; Jun 17; 9 (6) 10.1371/journal.pone.0099633 PMC4061032 24936866

[33] Yang SM, Ka SM, Wu HL, Yeh YC, Kuo CH, Hua KF, et al Thrombomodulin domain 1 ameliorates diabetic nephropathy in mice via anti-NF-κB/NLRP3 inflamma some-mediated inflammation, enhancement of NRF2 antioxidant activity and inhibition of apoptosis. Diabetologia. 2014; 57: 424–434. 10.1007/s00125-013-3115-6 24317792

