# Supplementary figures and images for: Combination of Active Components of Xiexin Decoction Ameliorates Renal Fibrosis Through the Inhibition of NF-κB and TGF-β1/Smad Pathways in db/db Diabetic Mice
Source: PLoS One. 2015 Mar 24;10(3):e0122661. doi: 10.1371/journal.pone.0122661 (PMC4372382; doi:10.1371/journal.pone.0122661)

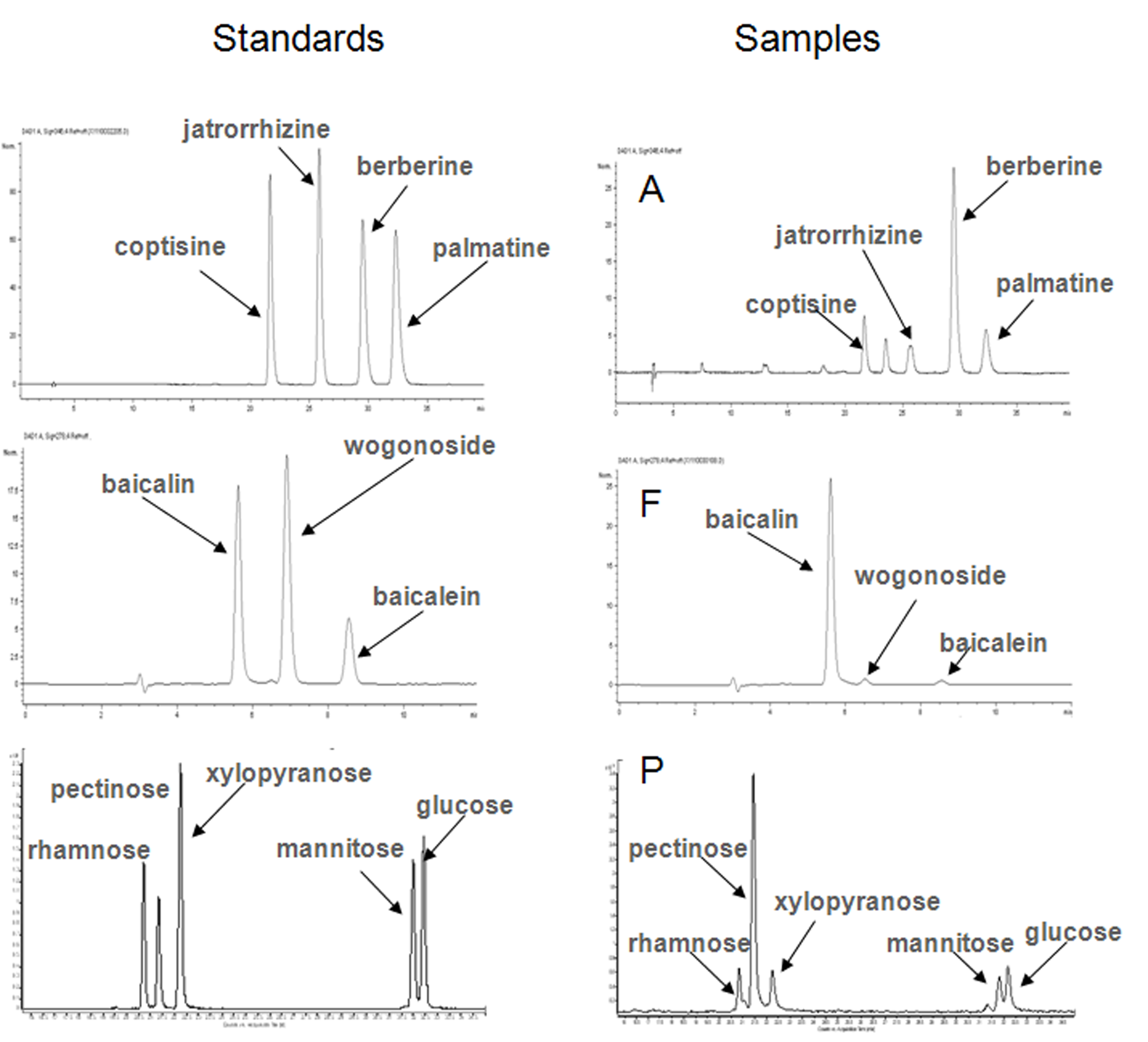

Supplement: S1 Fig — A and F were detected by HPLC. P was detected by GC. (TIF) [file pone.0122661.s001.tif]
